# A 30-Month Complete Urinary Obstruction Resulting from Trapped and Incarcerated Uterus: A Case Report

**DOI:** 10.3390/medicina57030207

**Published:** 2021-02-26

**Authors:** Gina Nam, Sa Ra Lee, Sung Hoon Kim, Hee Dong Chae

**Affiliations:** 1 Department of Obstetrics and Gynecology, Chung-Ang University Hospital, Chung-Ang University College of Medicine, 102, Heukseok-ro, Dongjak-gu, Seoul 06973, Korea; ginanam@caumc.or.kr; 2 Department of Obstetrics and Gynecology, University of Ulsan College of Medicine, Asan Medical Center, 88, Olympic-ro 43-gil, Songpa-gu, Seoul 05505, Korea; kimsung@amc.seoul.kr (S.H.K.); hdchae@amc.seoul.kr (H.D.C.)

**Keywords:** cervix, incarceration, myoma, urinary obstruction

## Abstract

Uterine incarceration is rare, but it can cause serious complications, in which the uterus is trapped in the pelvic cavity behind the sacral promontory. Fibroid uterus can cause urinary frequency and retention, which can result from compression of the urinary bladder with an enlarged fibroid uterus and the compression of the bladder neck or urethra, respectively. To our knowledge, there is no report on prolonged complete urinary obstruction because of an incarcerated uterus in nonpregnant women to date. A 51-year-old woman was referred for uterine myomas. She could not void for 30 months after she received an intradetrusor injection of botulinum toxin for urinary frequency management at the urology department of another hospital. She underwent clean intermittent catheterization for 30 months. She was referred to the gynecologic department for the evaluation of uterine myoma found on using abdominopelvic computed tomography. On physical examination, the uterine cervix was extremely displaced in the upward direction and was not exposed on speculum examination. Sonography and magnetic resonance imaging revealed that the urethra and the bladder neck were compressed by an extremely retroflexed fibroid uterus. Manual reduction of the incarcerated uterus failed; hence, we performed robot-assisted laparoscopic total hysterectomy with left salpingo-oophorectomy. She immediately urinated immediately after the operation and had normal urination at 1- and 48-month follow-up visits. Uterine incarceration by a fibroid uterus can cause complete urinary obstruction, as in this case. Uterine incarceration should be considered in women with urinary frequency or retention to avoid prolonged, serious complications.

## 1. Introduction

Approximately 15% of women have a retroflexed uterus [1]. If the uterus is enlarged and retroflexed, the fundus of the uterus can be entrapped between the sacral promontory and the pubic symphysis [2]. This condition usually results from pelvic adhesions because of a prior history of surgery, pelvic inflammatory disease, or endometriosis. The malformation of the uterus and the presence of fibroids increase the risk of uterine incarceration [3]. Patients present with nonspecific symptoms, such as abdominal pain, constipation, and urinary frequency or retention [3]. Acute urinary retention in women is common, and the most common cause of acute urinary retention is related to recent surgeries [4]. Although there are a few case reports on urinary retention with uterine incarceration, they usually occurred during pregnancy [2,5,6,7,8,9]. Uterine incarceration displaces the cervix anteriorly and compresses the urethra and the bladder neck. When uterine incarceration is clinically suspected, physical examination and imaging methods, such as sonography or magnetic resonance imaging (MRI), can confirm the diagnosis. Accurate diagnosis and treatment can prevent complications. Here, we present a rare case of uterine incarceration in a nonpregnant woman who presented with complete urinary obstruction and was successfully treated immediately after a hysterectomy. Intermittent urinary obstruction may sometimes occur, but there has never been reported such a complete urinary obstruction for a 30-month period.

## 2. Case Presentation

A 51-year-old perimenopausal woman was referred from the urology department for uterine myoma observed on abdominopelvic computed tomography. Thirty months previously, she complained of urinary frequency and was diagnosed as having overactive bladder (OAB) at the urology department of another hospital. Immediately after receiving a single intradetrusor injection of botulinum toxin (Botox^®^ 100 IU; Allergan Ltd., Madison, NJ, USA) to relieve OAB symptoms, she was unable to void despite having a voiding sense. She underwent clean intermittent catheterization (CIC) every 4 h for 30 months. Persistent urinary retention cannot be related to botulinum toxin injection because reinjection is required every 6 months for the management of overactive bladder [10]. She was depressed and attempted suicide 10 times because of this condition. She was diagnosed with acontractile bladder based on urodynamic study findings. However, she was referred to Obstetrics and Gynecology department for the extrinsic protruding mass and uterine myoma, which were observed using cystoscopy and abdominopelvic computed tomography, respectively.

**Figure 1:**
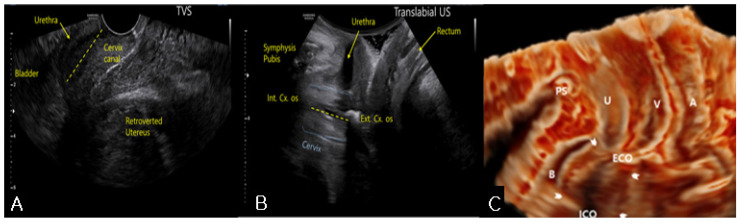
(**A**) Transvaginal sonography revealed an 18 × 7-cm sized, retroverted fibroid uterus and an elongated uterine cervix compressing the bladder neck; (**B**) Translabial sonography showed that the urethra and the bladder neck were compressed by extremely retroverted cervix; (**C**) Three-dimensional translabial sonography also demonstrated the compression of the urethra and the bladder neck (arrowhead). The elongated, ventrocranially displaced uterine cervix was compressing the urinary bladder. PS, pubis symphysis; B, Bladder; ECO, external cervical os; ICO, internal cervical os; V, vagina; A, rectum.

**Figure 2:**
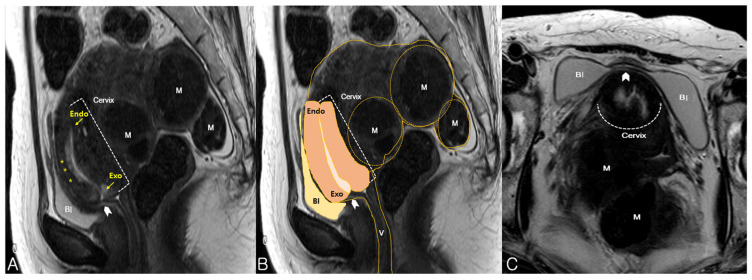
Magnetic resonance imaging findings. (**A**) Adenomyotic uterus with multiple myomas (M). The cervix was elongated and displaced ventrocranially, and compressed (arrowhead) the neck of the urinary bladder (Bl), as observed on mid-sagittal view; Elongated endocervical canal with high signal intensity is clearly visible with endocervical glands (asterisks); (**B**) A mid-sagittal view revealed an 18 × 7-cm sized, retroverted uterus with myomas (M), trapped in the pelvis. The urinary bladder (Bl) was compressed by the elongated cervix and vagina (V); (**C**) An axial view revealed a deformed bladder shape (arrowhead), dumbbell-shaped in this case, a clue that can be helpful in detecting an incarceration. Endo, endocervix; Exo, exocervix.

On pelvic examination, the uterine cervix was displaced anteriorly. Therefore, the cervix was out of reach and could not be exposed on vaginal examination. Sonography revealed an 18 × 7-cm retroflexed fibroid uterus and an elongated uterine cervix (Figure 1A). Translabial sonography revealed that the urethra and the bladder neck were compressed by an extremely retroverted cervix (Figure 1B). In most cases, the axis of the cervix is usually perpendicular to the sonographic probe, but in this case, it was nearly horizontal to the translabial sonographic probe. The elongated ventrocranially displaced uterine cervix was compressing the urinary bladder. MRI showed this relationship between the cervix and the bladder more clearly by revealing an elongated cervix compressing the urinary bladder neck (Figure 2A) as the incarcerated uterus lifted the cervix and fixed it. In addition, the bladder, which was hourglass-shaped, was completely compressed by the cervix (Figure 2C). Manual reduction for the management of the incarcerated uterus was unsuccessful. However, the patient was able to self-void several times immediately after the procedure, clearly suggesting that the prolonged urinary obstruction was caused by the incarceration. Thus, we performed a hysterectomy to treat the uterine incarceration.

**Figure 3:**
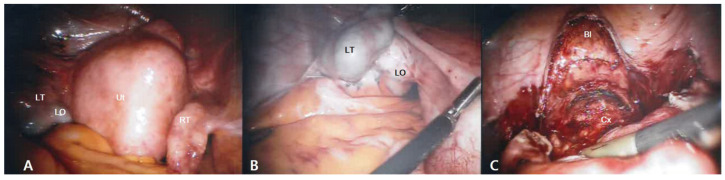
Robot-assisted laparoscopic view. (**A**) The fibroid Ut and both adnexae without adhesion between the anterior peritoneum and the uterus were noted; (**B**) The LT and LO were adhered together, and endometriosis spots and filmy adhesions were noted on the surface of the LO. There were no adhesions between the uterus and the posterior peritoneum; (**C**) Dissecting the bladder peritoneum revealed that there was no dense adhesion between the Bl and the Cx. LT, left fallopian tube; LO, left ovary; RT, right fallopian tube; Cx, uterine cervix; Ut, uterus; Bl, bladder.

We performed a robot-assisted laparoscopic total hysterectomy with a left salpingo-oophorectomy. Endometriosis and adhesion bands were noted on the surface of the left ovary and fallopian tube (Figure 3B), but no adhesions were noted between the uterus and the bladder (Figure 3C). Hence, it was not an adhesion-related issue; rather, it resulted from the action of the lever principle. The patient urinated normally immediately after the removal of the urethral Foley catheter 2 days after the operation. The patient maintained normal urination at the 1- and 48-month follow-up visits.

## 3. Discussion

There are several reports on the incarceration of the gravid uterus, which is known to be a rare but severe complication of pregnancy [2,5,6,7,8,9]. Although cases of the incarceration of a pedunculated uterine fibroid in an umbilical hernia in nonpregnant women have been reported [11], this challenges our current knowledge that uterine incarceration in the pelvis occurs only in a gravid uterus.

Factors that may predispose the patient to develop an incarcerated uterus include a history of pelvic inflammatory disease, prior gynecological surgery, endometriosis, uterine malformations, and uterine tumors, such as leiomyoma [2]. The development of acute urinary symptoms in patients with uterine leiomyoma is associated with fundal or posterior fibroids with a retroverted uterus [12].

Uterine incarceration diagnosis based on clinical presentation is difficult because the symptoms are not specific. The typical symptoms are genitourinary and gastrointestinal complaints associated with mechanical compression of the surrounding organs. Urinary retention can result from mechanical compression of the urethra and the bladder neck by the anteriorly displaced cervix under the symphysis pubis, as in this case. When the patient presents with urinary retention, even when gynecologic causes of urinary retention are unusual, a detailed patient history should be recorded, and abdominal and pelvic examinations should be performed at the first visit. The underlying disorder of urinary retention is usually detrusor failure rather than outflow obstruction [13]. However, reversible causes, such as fibroids and pelvic organ prolapse, should be considered, and a complete evaluation needs to be performed, including pelvic examination and ultrasonography. On physical examination, a retroflexed uterus in the posterior cul-de-sac is palpable, and the cervix is not visualized on speculum examination because it is anteriorly displaced. When uterine incarceration is suspected, targeted ultrasonography and MRI can aid early intervention. An elongated cervix above the symphysis pubis can be difficult to localize on transvaginal sonography; hence, transabdominal ultrasound examination is more accurate in revealing the cervix position and the relationship among the bladder, uterus, and vagina [14]. Translabial sonography can demonstrate uterine prolapse, enterocele, and rectocele, which can compress the urethra [15]. Hence, we utilized this clinical application of translabial sonography in the workup of the incarcerated, retroverted fibroid uterus with distortion or compression of the bladder neck by the cervix. MRI also shows a retroflexed uterus with the fundus below the sacral promontory and a cranial and ventral position of the cervix. The vagina is displaced almost parallel to the dorsal spine and has accompanying abnormalities, such as leiomyomas and endometriosis [16]. These MRI findings and the case description could be helpful in increasing awareness of the possibility of uterine incarceration when a woman presents with acute urinary retention in a nonpregnant condition.

A long delay in the recognition and treatment of uterine incarceration is associated with more complications. Infections of uterine contents, urinary tract infections, and rectal gangrene can occur [17]. Acute urinary retention may develop into bilateral hydronephrosis and bladder rupture [17,18]. Newell et al. [5] reported a case complicated by bladder atony resulting from a prolonged period of urinary retention before indwelling catheterization. Intermittent self-catheterization can be an option. Unfortunately, this case was initially misdiagnosed as an OAB, and after injection of botulinum toxin to relieve the relevant symptoms, the incarcerated uterus caused complete urinary obstruction.

Foley decompression of the bladder followed by manual reduction of the retroverted uterus by pushing the posterior cul-de-sac upward should be considered as a first-line treatment. Trendelenburg, knee-chest, or an all-fours position, and the use of a tenaculum are suggested to correct the uterus [5,7]. After successful manual reduction, a pessary can be used to remain in the pelvis and prevent recurrence [3,19]. If the aforementioned procedure is not successful, colonoscopic gas insufflation or, if indicated, hysterotomy delivery or hysterectomy in cases of a gravid or nongravid uterus, respectively, can be performed [7]. Sutter et al. [20] suggested an anterior fixation of the uterus at the time of cesarean delivery for their case, but the incarceration of the uterus recurred.

We searched all studies published from January 1986 to January 2021 using the terms “Uterine incarceration” and “Urinary retention” on PubMed. We found 26 reports of incarcerated gravid uterus with urinary retention. However, only two reports illustrated the acute presentation of uterine incarceration in nongravid women.

## 4. Conclusions

To our knowledge, this is the first report on the incarcerated uterus in a nonpregnant woman who had serious complications of prolonged complete urinary obstruction.

Physicians, especially gynecologists, urologists, emergency medical doctors, and radiologists, should consider the possibility of an incarcerated uterus when patients present with acute urinary retention and a retroverted uterus with an elongated upward displaced cervix. Manual reduction may provide a clue to the diagnosis, and hysterectomy can be a definite treatment to achieve a complete urinary obstruction and avoid long-term self-CIC, as in this case.

## Data Availability

Not applicable.
